# A DNA-barcoded Luria-Delbrück assay resolves mechanisms of adaptation

**DOI:** 10.64898/2026.08.03.742544

**Published:** 2026-08-07

**Authors:** Kabir Husain, LaNijah Flagg, David Pincus, Arvind Murugan

## Abstract

When populations encounter a harsh new environment, they adapt in many different ways – from spontaneous genetic changes to transient non-genetic plasticity. Distinguishing between mechanisms of adaptation is often difficult, as the key events are rare and hard to observe directly. Here, we introduce HiDenSeq, a Luria-Delbrück assay that uses DNA barcoding and deep sequencing to resolve the Luria-Delbrück distribution – the statistics of adaptation to a new environment – over four orders of magnitude. At this statistical depth, the shape of the distribution encodes the underlying adaptive mechanism. We find that DNA mismatch repair mutants shift the distribution’s scale without changing its shape, indicating their only effect is as mutator alleles. A pulse of UV mutagenesis, however, adds a second, statistically distinguishable mode atop the spontaneous background, showing that different mechanisms of adaptation can be quantified directly from the distribution. By sampling rare-event statistics with DNA barcodes, HiDenSeq provides a general method for studying mechanisms of adaptation in evolving populations from microbes to cancers.

## Full Text Availability

The license terms selected by the author(s) for this preprint version do not permit archiving in PMC. The full text is available from the preprint server.

